# Cinnarizinium picrate

**DOI:** 10.1107/S1600536812020764

**Published:** 2012-05-16

**Authors:** Yanxi Song, C. S. Chidan Kumar, G. B. Nethravathi, S. Naveen, Hongqi Li

**Affiliations:** a School of Environmental Science and Engineering, Donghua University, Shanghai 201620, People’s Republic of China; b Department of Chemistry, G. Madegowda Institute of Technology, Bharathi Nagar 571 422, India; c Department of Chemistry, B.E.T. Academy of Higher Education, Bharathi Nagar 571 422, India; d Department of Physics, School of Engineering and Technology, Jain University, Bangalore 562 112, India; e Key Laboratory of Science & Technology of Eco-Textiles, Ministry of Education, College of Chemistry, Chemical Engineering & Biotechnology, Donghua University, Shanghai 201620, People’s Republic of China

## Abstract

In the title salt {systematic name: 4-diphenyl­methyl-1-[(*E*)-3-phenyl­prop-2-en-1-yl]piperazin-1-ium 2,4,6-trinitro­pheno­late), C_26_H_29_N_2_^+^·C_6_H_2_N_3_O_7_^−^, the cinnarizinium cation is protonated at the piperazine N atom connected to the styrenylmethyl group; the piperazine ring adopts a distorted chair conformaiton. In the crystal, bifurcated N—H⋯(O,O) hydrogen bonds link the components into two-ion aggregates.

## Related literature

For background to the anti-histamine cinnarizine, see: Towse (1980 ▶); Barrett & Zolov (1960 ▶). For related structures, see: Mouillé *et al.* (1975 ▶); Bertolasi *et al.* (1980 ▶); Jasinski *et al.* (2011 ▶). For additional conformational analysis, see: Cremer & Pople (1975 ▶).
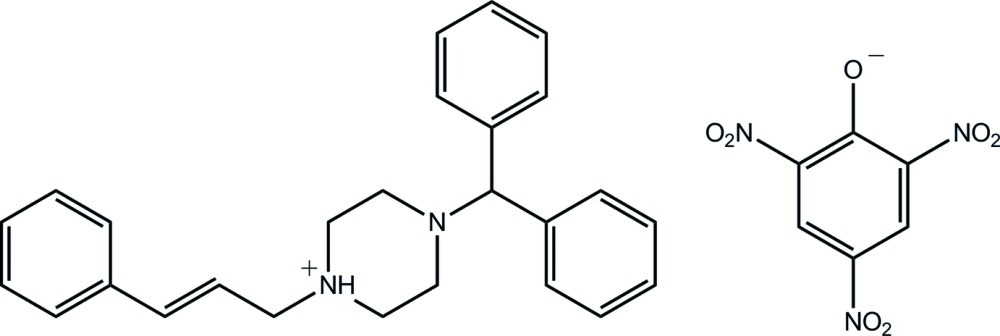


## Experimental

### 

#### Crystal data

C_26_H_29_N_2_^+^·C_6_H_2_N_3_O_7_^−^
*M* *_r_* = 597.62
Monoclinic, 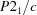

*a* = 14.5906 (19) Å
*b* = 12.7720 (17) Å
*c* = 16.441 (2) Å
β = 103.114 (2)°
*V* = 2984.0 (7) Å^3^
*Z* = 4
Mo *K*α radiation
μ = 0.10 mm^−1^
*T* = 296 K
0.16 × 0.16 × 0.07 mm

#### Data collection

Bruker APEXII CCD diffractometer
Absorption correction: multi-scan (*SADABS*; Sheldrick, 1997 ▶) *T*_min_ = 0.985, *T*_max_ = 0.993
15196 measured reflections
5262 independent reflections
3181 reflections with *I* > 2σ(*I*)
*R*_int_ = 0.033

#### Refinement

*R*[*F* ^2^ > 2σ(*F* ^2^)] = 0.051
*wR*(*F* ^2^) = 0.143
*S* = 1.03
5262 reflections
401 parameters
H atoms treated by a mixture of independent and constrained refinement
Δρ_max_ = 0.21 e Å^−3^
Δρ_min_ = −0.17 e Å^−3^

### 

Data collection: *APEX2* (Bruker, 2004 ▶); cell refinement: *SAINT* (Bruker, 2004 ▶); data reduction: *SAINT*; program(s) used to solve structure: *SHELXS97* (Sheldrick, 2008 ▶); program(s) used to refine structure: *SHELXL97* (Sheldrick, 2008 ▶); molecular graphics: *SHELXTL* (Sheldrick, 2008 ▶); software used to prepare material for publication: *SHELXTL*.

## Supplementary Material

Crystal structure: contains datablock(s) global, I. DOI: 10.1107/S1600536812020764/tk5091sup1.cif

Structure factors: contains datablock(s) I. DOI: 10.1107/S1600536812020764/tk5091Isup2.hkl

Supplementary material file. DOI: 10.1107/S1600536812020764/tk5091Isup3.cml

Additional supplementary materials:  crystallographic information; 3D view; checkCIF report

## Figures and Tables

**Table 1:** Hydrogen-bond geometry (Å, °)

*D*—H⋯*A*	*D*—H	H⋯*A*	*D*⋯*A*	*D*—H⋯*A*
N2—H2*A*⋯O7^i^	0.94 (3)	2.59 (2)	3.119 (3)	116.6 (18)
N2—H2*A*⋯O1^i^	0.94 (3)	1.79 (3)	2.710 (3)	168 (2)

Symmetry code: (i) 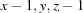.

**Fig. 1.**
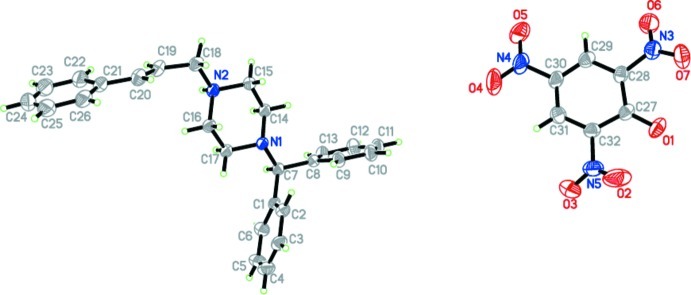
A view of the molecule structure of the title compound. Displacement ellipsoids are drawn at the 30% probability level.

## supplementary crystallographic information

### Comment

Cinnarizine (Stugeron, Stunarone) is an anti-histamine which is mainly used for the control of nausea and vomiting due to motion sickness. Cinnarizine could be also viewed as a nootropic drug because of its vasorelaxating abilities (due to calcium channel blockage) and as a labyrinthine sedative (Towse *et al.*, 1980). A clinical evaluation of cinnarizine in various allergic disorders has been reported earlier (Barrett *et al.*, 1960). Cinnarizine can be used in scuba divers without an increased risk of central nervous system oxygen toxicity. The crystal structures of some related compounds *viz*. cinnarizine (Mouillé *et al.*, 1975) and cyclizine hydrochloride (Bertolasi *et al.*, 1980) have been reported. In view of the above, and as a part of our studies on the salts of the piperazines, the title compound was synthesized and herein we report its crystal structure.

The molecular structure and atom numbering scheme of the title compound are shown in Fig 1. In the structure, the piperazine ring adopts a slightly distorted chair conformation with the puckering parameters Q, θ and φ having values of 0.584 (2)°, 174.3 (2)° and 179 (2)°, respectively (Cremer & Pople, 1975). These values slightly different from those reported earlier for cinnarizinium dipicrate (Jasinski *et al.*, 2011). For an ideal chair conformation, θ has a value of 0 or 180°. The sum of the bond angles around the piperazine-N atoms N1 and N2 are 328.94° and 332.45°, respectively, indicating that they are *sp*^3^ hybridized. The bonds N1—C7 and N2—C18 connecting the diphenylmethyl and the phenyl-but-2-ene groups make an angle of 74.44 (14)° and 70.28 (14)°, respectively, with the Cremer and Pople (1975) plane of the piperazine ring and thus the substituents are in the equatorial plane. The dihedral angle between the piperazine ring and the phenyl ring (C21–C26) bridged by the but-2-ene group is 63.50 (12)° whereas the dihedral angles between the piperazine ring and the diphenyl methyl rings (C1–C6) and (C8–C13) are 77.63 (11)° and 89.85 (15)°, respectively. In the crystal structure, N—H···O hydrogen bonds link the ions into two ion aggregates.

### Experimental

Cinnarizine (3.68 g, 0.01 mol) and picric acid (2.99 g, 0.01 mol) were dissolved separately in methanol. The solutions were mixed and stirred for a few minutes at room temperature. The precipitate was collected by filtration and purified by recrystallization from methanol. On recrystallization with DMF after 15 days, good quality single crystals were obtained; *M*.pt: 463–465 K.

### Refinement

All H atoms were placed at calculated positions and refined using a riding model approximation, with C—H distances in the range 0.93—0.98 Å, and with *U*_iso_(H) = 1.2*U*_eq_(C). The ammonium-H atom was refined freely.

### Crystal data

**Table:**

C_26_H_29_N_2_^+^·C_6_H_2_N_3_O_7_^−^	*F*(000) = 1256
*M**_r_* = 597.62	*D*_x_ = 1.330 Mg m^−^^3^
Monoclinic, *P*2_1_/*c*	Melting point = 465–463 K
Hall symbol: -P 2ybc	Mo *K*α radiation, λ = 0.71073 Å
*a* = 14.5906 (19) Å	Cell parameters from 2307 reflections
*b* = 12.7720 (17) Å	θ = 2.3–22.0°
*c* = 16.441 (2) Å	µ = 0.10 mm^−^^1^
β = 103.114 (2)°	*T* = 296 K
*V* = 2984.0 (7) Å^3^	Block, yellow
*Z* = 4	0.16 × 0.16 × 0.07 mm

### Data collection

**Table:**

Bruker APEXII CCD diffractometer	5262 independent reflections
Radiation source: fine-focus sealed tube	3181 reflections with *I* > 2σ(*I*)
Graphite monochromator	*R*_int_ = 0.033
φ and ω scans	θ_max_ = 25.1°, θ_min_ = 2.0°
Absorption correction: multi-scan (*SADABS*; Sheldrick, 1997)	*h* = −14→17
*T*_min_ = 0.985, *T*_max_ = 0.993	*k* = −15→15
15196 measured reflections	*l* = −19→15

### Refinement

**Table:**

Refinement on *F*^2^	Primary atom site location: structure-invariant direct methods
Least-squares matrix: full	Secondary atom site location: difference Fourier map
*R*[*F*^2^ > 2σ(*F*^2^)] = 0.051	Hydrogen site location: inferred from neighbouring sites
*wR*(*F*^2^) = 0.143	H atoms treated by a mixture of independent and constrained refinement
*S* = 1.03	*w* = 1/[σ^2^(*F*_o_^2^) + (0.0606*P*)^2^ + 0.4273*P*] where *P* = (*F*_o_^2^ + 2*F*_c_^2^)/3
5262 reflections	(Δ/σ)_max_ < 0.001
401 parameters	Δρ_max_ = 0.21 e Å^−^^3^
0 restraints	Δρ_min_ = −0.17 e Å^−^^3^

### Special details

**Table:**

Geometry. All e.s.d.'s (except the e.s.d. in the dihedral angle between two l.s. planes) are estimated using the full covariance matrix. The cell e.s.d.'s are taken into account individually in the estimation of e.s.d.'s in distances, angles and torsion angles; correlations between e.s.d.'s in cell parameters are only used when they are defined by crystal symmetry. An approximate (isotropic) treatment of cell e.s.d.'s is used for estimating e.s.d.'s involving l.s. planes.
Refinement. Refinement of *F*^2^ against ALL reflections. The weighted *R*-factor *wR* and goodness of fit *S* are based on *F*^2^, conventional *R*-factors *R* are based on *F*, with *F* set to zero for negative *F*^2^. The threshold expression of *F*^2^ > σ(*F*^2^) is used only for calculating *R*-factors(gt) *etc*. and is not relevant to the choice of reflections for refinement. *R*-factors based on *F*^2^ are statistically about twice as large as those based on *F*, and *R*- factors based on ALL data will be even larger.

### Fractional atomic coordinates and isotropic or equivalent isotropic displacement parameters (Å^2^)

**Table:**

	*x*	*y*	*z*	*U*_iso_*/*U*_eq_	
C1	0.52622 (15)	1.01467 (17)	0.19638 (13)	0.0431 (5)	
C2	0.59492 (16)	0.93827 (18)	0.21939 (14)	0.0507 (6)	
H2	0.5911	0.8916	0.2619	0.061*	
C3	0.66912 (17)	0.9304 (2)	0.18004 (17)	0.0616 (7)	
H3	0.7152	0.8796	0.1968	0.074*	
C4	0.67436 (19)	0.9977 (2)	0.11638 (18)	0.0688 (8)	
H4	0.7240	0.9924	0.0898	0.083*	
C5	0.6071 (2)	1.0725 (2)	0.09190 (16)	0.0714 (8)	
H5	0.6107	1.1175	0.0483	0.086*	
C6	0.53323 (18)	1.0819 (2)	0.13170 (15)	0.0596 (7)	
H6	0.4880	1.1336	0.1149	0.072*	
C7	0.44424 (14)	1.02478 (16)	0.23909 (13)	0.0427 (5)	
H7	0.3976	1.0715	0.2049	0.051*	
C8	0.47390 (15)	1.07319 (17)	0.32545 (13)	0.0425 (5)	
C9	0.54277 (16)	1.02889 (19)	0.38803 (14)	0.0535 (6)	
H9	0.5714	0.9667	0.3778	0.064*	
C10	0.56926 (18)	1.0761 (2)	0.46534 (15)	0.0637 (7)	
H10	0.6164	1.0461	0.5064	0.076*	
C11	0.52685 (19)	1.1666 (2)	0.48211 (16)	0.0638 (7)	
H11	0.5447	1.1979	0.5344	0.077*	
C12	0.45798 (19)	1.2107 (2)	0.42127 (16)	0.0666 (7)	
H12	0.4283	1.2718	0.4324	0.080*	
C13	0.43233 (17)	1.16465 (18)	0.34333 (15)	0.0575 (7)	
H13	0.3862	1.1960	0.3022	0.069*	
C14	0.31810 (15)	0.92742 (18)	0.27987 (13)	0.0478 (6)	
H14A	0.2709	0.9733	0.2470	0.057*	
H14B	0.3377	0.9570	0.3355	0.057*	
C15	0.27606 (16)	0.82031 (18)	0.28533 (13)	0.0510 (6)	
H15A	0.3223	0.7757	0.3208	0.061*	
H15B	0.2223	0.8264	0.3106	0.061*	
C16	0.32487 (15)	0.77316 (18)	0.15764 (14)	0.0507 (6)	
H16A	0.3026	0.7485	0.1008	0.061*	
H16B	0.3740	0.7260	0.1859	0.061*	
C17	0.36469 (15)	0.88150 (18)	0.15634 (13)	0.0496 (6)	
H17A	0.4160	0.8801	0.1278	0.060*	
H17B	0.3164	0.9280	0.1257	0.060*	
C18	0.20741 (18)	0.66271 (18)	0.20475 (15)	0.0573 (6)	
H18A	0.2580	0.6161	0.2309	0.069*	
H18B	0.1609	0.6630	0.2385	0.069*	
C19	0.16375 (17)	0.62381 (19)	0.11991 (16)	0.0589 (7)	
H19	0.1098	0.6581	0.0912	0.071*	
C20	0.19386 (17)	0.5462 (2)	0.08192 (16)	0.0634 (7)	
H20	0.2454	0.5100	0.1128	0.076*	
C21	0.15677 (19)	0.5088 (2)	−0.00331 (16)	0.0634 (7)	
C22	0.0751 (2)	0.5494 (2)	−0.05376 (18)	0.0794 (9)	
H22	0.0409	0.5996	−0.0322	0.095*	
C23	0.0439 (3)	0.5168 (3)	−0.1347 (2)	0.1061 (13)	
H23	−0.0114	0.5440	−0.1675	0.127*	
C24	0.0947 (4)	0.4441 (4)	−0.1668 (2)	0.1206 (17)	
H24	0.0742	0.4231	−0.2221	0.145*	
C25	0.1749 (3)	0.4018 (3)	−0.1191 (3)	0.1079 (13)	
H25	0.2086	0.3520	−0.1415	0.129*	
C26	0.2056 (2)	0.4338 (2)	−0.0368 (2)	0.0829 (9)	
H26	0.2597	0.4044	−0.0038	0.099*	
C27	1.04152 (17)	0.86358 (18)	1.05005 (17)	0.0566 (7)	
C28	0.95804 (17)	0.84392 (19)	1.08034 (16)	0.0564 (6)	
C29	0.87055 (17)	0.8270 (2)	1.02945 (17)	0.0631 (7)	
H29	0.8182	0.8190	1.0523	0.076*	
C30	0.86144 (18)	0.8220 (2)	0.94491 (17)	0.0639 (7)	
C31	0.93837 (19)	0.8327 (2)	0.91018 (17)	0.0682 (8)	
H31	0.9317	0.8274	0.8527	0.082*	
C32	1.02445 (17)	0.85131 (19)	0.96112 (17)	0.0589 (7)	
N1	0.39904 (11)	0.92136 (13)	0.24143 (10)	0.0418 (4)	
N2	0.24573 (13)	0.77163 (15)	0.20113 (11)	0.0458 (5)	
N3	0.96429 (18)	0.83694 (19)	1.16985 (15)	0.0744 (7)	
N4	0.77007 (19)	0.8000 (2)	0.89112 (19)	0.0919 (8)	
N5	1.10421 (19)	0.8585 (2)	0.92183 (18)	0.0847 (8)	
O1	1.11914 (12)	0.89116 (14)	1.09451 (12)	0.0783 (6)	
O2	1.17777 (18)	0.8186 (2)	0.95562 (19)	0.1361 (11)	
O3	1.09238 (17)	0.9046 (2)	0.85521 (15)	0.1185 (9)	
O4	0.76491 (17)	0.7930 (2)	0.81627 (17)	0.1279 (10)	
O5	0.70246 (16)	0.7909 (2)	0.92284 (17)	0.1272 (10)	
O6	0.89418 (16)	0.8534 (2)	1.19597 (13)	0.1050 (8)	
O7	1.03892 (16)	0.81087 (19)	1.21524 (12)	0.1012 (8)	
H2A	0.1979 (18)	0.8141 (19)	0.1706 (15)	0.071 (8)*	

### Atomic displacement parameters (Å^2^)

**Table:**

	*U*^11^	*U*^22^	*U*^33^	*U*^12^	*U*^13^	*U*^23^
C1	0.0374 (12)	0.0451 (13)	0.0457 (13)	−0.0046 (10)	0.0073 (10)	−0.0056 (10)
C2	0.0455 (14)	0.0493 (14)	0.0577 (15)	−0.0011 (11)	0.0124 (11)	−0.0023 (12)
C3	0.0470 (15)	0.0614 (16)	0.0783 (18)	−0.0019 (13)	0.0180 (14)	−0.0155 (15)
C4	0.0629 (18)	0.0650 (18)	0.089 (2)	−0.0131 (15)	0.0395 (16)	−0.0199 (16)
C5	0.087 (2)	0.0680 (18)	0.0700 (18)	−0.0081 (17)	0.0415 (16)	0.0043 (15)
C6	0.0658 (17)	0.0579 (16)	0.0580 (16)	0.0000 (13)	0.0199 (13)	0.0037 (13)
C7	0.0376 (12)	0.0453 (13)	0.0440 (13)	0.0005 (10)	0.0069 (10)	0.0003 (10)
C8	0.0412 (12)	0.0425 (12)	0.0436 (13)	−0.0055 (10)	0.0092 (10)	−0.0015 (10)
C9	0.0498 (14)	0.0611 (15)	0.0471 (14)	0.0071 (12)	0.0058 (11)	−0.0013 (12)
C10	0.0603 (17)	0.0786 (19)	0.0466 (15)	0.0038 (14)	0.0002 (12)	0.0007 (13)
C11	0.0782 (19)	0.0606 (17)	0.0502 (15)	−0.0116 (15)	0.0097 (14)	−0.0115 (13)
C12	0.084 (2)	0.0510 (15)	0.0606 (17)	0.0058 (14)	0.0072 (15)	−0.0100 (13)
C13	0.0625 (16)	0.0507 (14)	0.0543 (15)	0.0088 (12)	0.0026 (12)	−0.0052 (12)
C14	0.0430 (13)	0.0548 (14)	0.0463 (13)	−0.0033 (11)	0.0117 (11)	−0.0078 (11)
C15	0.0471 (14)	0.0617 (15)	0.0447 (13)	−0.0070 (12)	0.0113 (11)	−0.0056 (11)
C16	0.0409 (13)	0.0591 (15)	0.0533 (14)	−0.0048 (11)	0.0132 (11)	−0.0115 (12)
C17	0.0404 (13)	0.0605 (15)	0.0490 (14)	−0.0080 (11)	0.0124 (11)	−0.0116 (11)
C18	0.0587 (15)	0.0462 (14)	0.0661 (17)	−0.0088 (12)	0.0120 (13)	0.0013 (12)
C19	0.0508 (15)	0.0489 (14)	0.0696 (17)	−0.0067 (12)	−0.0019 (13)	−0.0012 (13)
C20	0.0555 (16)	0.0620 (17)	0.0680 (17)	−0.0004 (13)	0.0041 (13)	−0.0009 (14)
C21	0.0639 (17)	0.0642 (17)	0.0604 (17)	−0.0202 (14)	0.0104 (14)	−0.0037 (14)
C22	0.079 (2)	0.089 (2)	0.0653 (19)	−0.0205 (17)	0.0050 (16)	0.0063 (16)
C23	0.109 (3)	0.136 (3)	0.064 (2)	−0.053 (3)	−0.001 (2)	0.021 (2)
C24	0.156 (4)	0.143 (4)	0.065 (2)	−0.085 (4)	0.031 (3)	−0.023 (3)
C25	0.132 (4)	0.106 (3)	0.101 (3)	−0.051 (3)	0.057 (3)	−0.033 (2)
C26	0.086 (2)	0.074 (2)	0.092 (2)	−0.0284 (17)	0.0263 (18)	−0.0186 (18)
C27	0.0428 (15)	0.0475 (14)	0.0742 (18)	0.0036 (11)	0.0022 (13)	0.0119 (13)
C28	0.0469 (15)	0.0597 (16)	0.0597 (16)	0.0076 (12)	0.0061 (12)	−0.0017 (12)
C29	0.0436 (15)	0.0696 (18)	0.0748 (19)	0.0071 (13)	0.0110 (13)	0.0028 (14)
C30	0.0440 (15)	0.0724 (18)	0.0671 (18)	0.0045 (13)	−0.0044 (13)	0.0109 (14)
C31	0.0667 (19)	0.0738 (18)	0.0592 (17)	0.0046 (15)	0.0042 (15)	0.0179 (14)
C32	0.0489 (15)	0.0607 (16)	0.0679 (18)	0.0020 (12)	0.0148 (13)	0.0167 (13)
N1	0.0365 (10)	0.0470 (11)	0.0418 (10)	−0.0038 (8)	0.0084 (8)	−0.0065 (8)
N2	0.0396 (11)	0.0494 (12)	0.0470 (12)	−0.0025 (9)	0.0069 (9)	−0.0044 (9)
N3	0.0598 (16)	0.0925 (18)	0.0698 (17)	0.0040 (14)	0.0123 (14)	−0.0179 (13)
N4	0.0593 (18)	0.112 (2)	0.088 (2)	0.0053 (16)	−0.0179 (16)	0.0149 (17)
N5	0.0690 (18)	0.098 (2)	0.091 (2)	−0.0080 (15)	0.0273 (16)	0.0238 (16)
O1	0.0501 (11)	0.0683 (12)	0.1024 (15)	−0.0069 (9)	−0.0123 (10)	0.0134 (10)
O2	0.0716 (16)	0.177 (3)	0.174 (3)	0.0305 (17)	0.0569 (17)	0.071 (2)
O3	0.1133 (19)	0.164 (2)	0.0846 (16)	−0.0169 (17)	0.0360 (14)	0.0298 (17)
O4	0.0950 (18)	0.180 (3)	0.0842 (17)	0.0107 (17)	−0.0297 (14)	−0.0095 (18)
O5	0.0481 (13)	0.188 (3)	0.131 (2)	−0.0046 (16)	−0.0087 (14)	0.0326 (19)
O6	0.0751 (15)	0.160 (2)	0.0868 (15)	0.0011 (15)	0.0321 (13)	−0.0238 (14)
O7	0.0840 (15)	0.152 (2)	0.0609 (13)	0.0327 (15)	0.0013 (11)	−0.0123 (13)

### Geometric parameters (Å, º)

**Table:**

C1—C6	1.389 (3)	C17—H17B	0.9700
C1—C2	1.388 (3)	C18—C19	1.482 (3)
C1—C7	1.524 (3)	C18—N2	1.506 (3)
C2—C3	1.385 (3)	C18—H18A	0.9700
C2—H2	0.9300	C18—H18B	0.9700
C3—C4	1.370 (3)	C19—C20	1.300 (3)
C3—H3	0.9300	C19—H19	0.9300
C4—C5	1.364 (4)	C20—C21	1.463 (3)
C4—H4	0.9300	C20—H20	0.9300
C5—C6	1.387 (3)	C21—C26	1.381 (4)
C5—H5	0.9300	C21—C22	1.389 (4)
C6—H6	0.9300	C22—C23	1.369 (4)
C7—N1	1.481 (3)	C22—H22	0.9300
C7—C8	1.519 (3)	C23—C24	1.366 (6)
C7—H7	0.9800	C23—H23	0.9300
C8—C13	1.378 (3)	C24—C25	1.364 (6)
C8—C9	1.385 (3)	C24—H24	0.9300
C9—C10	1.380 (3)	C25—C26	1.387 (4)
C9—H9	0.9300	C25—H25	0.9300
C10—C11	1.369 (3)	C26—H26	0.9300
C10—H10	0.9300	C27—O1	1.251 (3)
C11—C12	1.368 (3)	C27—C32	1.435 (3)
C11—H11	0.9300	C27—C28	1.439 (3)
C12—C13	1.382 (3)	C28—C29	1.375 (3)
C12—H12	0.9300	C28—N3	1.456 (3)
C13—H13	0.9300	C29—C30	1.367 (3)
C14—N1	1.462 (3)	C29—H29	0.9300
C14—C15	1.510 (3)	C30—C31	1.377 (4)
C14—H14A	0.9700	C30—N4	1.450 (3)
C14—H14B	0.9700	C31—C32	1.363 (3)
C15—N2	1.490 (3)	C31—H31	0.9300
C15—H15A	0.9700	C32—N5	1.456 (3)
C15—H15B	0.9700	N2—H2A	0.94 (3)
C16—N2	1.490 (3)	N3—O6	1.214 (3)
C16—C17	1.503 (3)	N3—O7	1.218 (3)
C16—H16A	0.9700	N4—O4	1.219 (3)
C16—H16B	0.9700	N4—O5	1.222 (3)
C17—N1	1.466 (2)	N5—O2	1.204 (3)
C17—H17A	0.9700	N5—O3	1.221 (3)
			
C6—C1—C2	118.0 (2)	C19—C18—N2	110.90 (19)
C6—C1—C7	120.2 (2)	C19—C18—H18A	109.5
C2—C1—C7	121.8 (2)	N2—C18—H18A	109.5
C3—C2—C1	121.1 (2)	C19—C18—H18B	109.5
C3—C2—H2	119.5	N2—C18—H18B	109.5
C1—C2—H2	119.5	H18A—C18—H18B	108.0
C4—C3—C2	119.7 (2)	C20—C19—C18	126.0 (2)
C4—C3—H3	120.1	C20—C19—H19	117.0
C2—C3—H3	120.1	C18—C19—H19	117.0
C5—C4—C3	120.3 (3)	C19—C20—C21	128.1 (3)
C5—C4—H4	119.9	C19—C20—H20	115.9
C3—C4—H4	119.9	C21—C20—H20	115.9
C4—C5—C6	120.4 (3)	C26—C21—C22	118.1 (3)
C4—C5—H5	119.8	C26—C21—C20	119.7 (3)
C6—C5—H5	119.8	C22—C21—C20	122.1 (3)
C5—C6—C1	120.5 (2)	C23—C22—C21	121.2 (3)
C5—C6—H6	119.7	C23—C22—H22	119.4
C1—C6—H6	119.7	C21—C22—H22	119.4
N1—C7—C8	111.90 (16)	C24—C23—C22	119.5 (4)
N1—C7—C1	109.71 (17)	C24—C23—H23	120.2
C8—C7—C1	112.23 (17)	C22—C23—H23	120.3
N1—C7—H7	107.6	C25—C24—C23	121.1 (4)
C8—C7—H7	107.6	C25—C24—H24	119.4
C1—C7—H7	107.6	C23—C24—H24	119.4
C13—C8—C9	117.9 (2)	C24—C25—C26	119.3 (4)
C13—C8—C7	119.9 (2)	C24—C25—H25	120.4
C9—C8—C7	122.2 (2)	C26—C25—H25	120.4
C10—C9—C8	120.7 (2)	C21—C26—C25	120.8 (3)
C10—C9—H9	119.7	C21—C26—H26	119.6
C8—C9—H9	119.7	C25—C26—H26	119.6
C11—C10—C9	120.6 (2)	O1—C27—C32	123.4 (3)
C11—C10—H10	119.7	O1—C27—C28	124.9 (3)
C9—C10—H10	119.7	C32—C27—C28	111.6 (2)
C12—C11—C10	119.4 (2)	C29—C28—C27	124.0 (2)
C12—C11—H11	120.3	C29—C28—N3	116.2 (2)
C10—C11—H11	120.3	C27—C28—N3	119.8 (2)
C11—C12—C13	120.2 (2)	C30—C29—C28	119.2 (3)
C11—C12—H12	119.9	C30—C29—H29	120.4
C13—C12—H12	119.9	C28—C29—H29	120.4
C8—C13—C12	121.2 (2)	C29—C30—C31	121.1 (2)
C8—C13—H13	119.4	C29—C30—N4	119.5 (3)
C12—C13—H13	119.4	C31—C30—N4	119.3 (3)
N1—C14—C15	110.89 (18)	C32—C31—C30	119.1 (3)
N1—C14—H14A	109.5	C32—C31—H31	120.4
C15—C14—H14A	109.5	C30—C31—H31	120.4
N1—C14—H14B	109.5	C31—C32—C27	124.6 (3)
C15—C14—H14B	109.5	C31—C32—N5	117.1 (3)
H14A—C14—H14B	108.0	C27—C32—N5	118.3 (2)
N2—C15—C14	111.20 (18)	C14—N1—C17	107.22 (16)
N2—C15—H15A	109.4	C14—N1—C7	111.85 (16)
C14—C15—H15A	109.4	C17—N1—C7	110.05 (16)
N2—C15—H15B	109.4	C16—N2—C15	109.96 (17)
C14—C15—H15B	109.4	C16—N2—C18	111.49 (18)
H15A—C15—H15B	108.0	C15—N2—C18	112.54 (18)
N2—C16—C17	111.29 (18)	C16—N2—H2A	107.5 (15)
N2—C16—H16A	109.4	C15—N2—H2A	106.5 (15)
C17—C16—H16A	109.4	C18—N2—H2A	108.5 (15)
N2—C16—H16B	109.4	O6—N3—O7	122.6 (3)
C17—C16—H16B	109.4	O6—N3—C28	118.8 (2)
H16A—C16—H16B	108.0	O7—N3—C28	118.6 (2)
N1—C17—C16	110.84 (18)	O4—N4—O5	123.6 (3)
N1—C17—H17A	109.5	O4—N4—C30	117.9 (3)
C16—C17—H17A	109.5	O5—N4—C30	118.5 (3)
N1—C17—H17B	109.5	O2—N5—O3	123.2 (3)
C16—C17—H17B	109.5	O2—N5—C32	119.1 (3)
H17A—C17—H17B	108.1	O3—N5—C32	117.6 (3)

### Hydrogen-bond geometry (Å, º)

**Table:**

*D*—H···*A*	*D*—H	H···*A*	*D*···*A*	*D*—H···*A*
N2—H2*A*···O7^i^	0.94 (3)	2.59 (2)	3.119 (3)	116.6 (18)
N2—H2*A*···O1^i^	0.94 (3)	1.79 (3)	2.710 (3)	168 (2)

Symmetry code: (i) *x*−1, *y*, *z*−1.
